# Hematological and biochemical markers and cytokine levels in hospitalized psychiatric patients with COVID-19

**DOI:** 10.3389/fimmu.2025.1536117

**Published:** 2025-04-11

**Authors:** Huirong Dai, Chih-Jung Chang, Zishun Li, Farong Liu, Qiao Zhang, Yixuan Bai, Pan You

**Affiliations:** ^1^ Xiamen Xianyue Hospital, Xianyue Hospital Affiliated to Xiamen Medical College, Fujian Psychiatric Center, Fujian Clinical Research Center for Mental Disorders, Xiamen, Fujian, China; ^2^ School of Medicine, Huaqiao University, Quanzhou, Fujian, China; ^3^ Medical Research Center, Xiamen Chang Gung Hospital, Xiamen, Fujian, China; ^4^ Xiamen Chang Gung Allergology Consortium, Xiamen Chang Gung Hospital, Xiamen, Fujian, China; ^5^ Department of Clinical Laboratory, The Third Hospital of Xiamen, Xiamen, Fujian, China

**Keywords:** COVID-19, psychiatric diseases, neutrophil-to-lymphocyte ratio, monocyte-to-lymphocyte ratio, systemic immune-inflammation index, C-reactive protein, magnesium, interleukin 6

## Abstract

**Background:**

Multiple lines of evidence indicate a connection between the pathogenesis of coronavirus disease 2019 (COVID-19) and psychiatric diseases (PDs). To improve the treatment and management of individuals with psychosis and COVID-19, we evaluated biomarkers of PD patients, including those with schizophrenia (SCZ), bipolar disorder (BD), and major depression (MDD), along with the biomarkers of COVID-19.

**Methods:**

In this study, 104 inpatients with concurrent PD and COVID-19 (PD+), the same 104 PD patients after they had recovered from COVID-19 (PD-), and 97 healthy controls (HCs) were evaluated. We analyzed the peripheral blood hematological parameters, serum biochemical parameters, and cytokine levels of the participants and compared the results among the three groups.

**Results:**

The monocyte count; neutrophil-to-lymphocyte ratio (NLR); monocyte-to-lymphocyte ratio (MLR); systemic immune-inflammation index (SII); and C-reactive protein (CRP), serum CK isoenzyme MB (CK-MB), glucose (GLU), and interleukin (IL)-6 levels were significantly greater (*P* < 0.05), whereas the magnesium (Mg) level was lower (*P* < 0.05) in both the PD+ and PD- groups than in the HC group. Moreover, the above indicators were significantly different between the PD+ and PD- groups (*P* < 0.05).

**Conclusion:**

Neutrophil count, monocyte count, NLR, MLR, SII, CRP, CK-MB, GLU and IL-6 levels were positively correlated with COVID-19 and PD. The Mg level was negatively correlated with COVID-19 and PD. Our findings suggest that Mg supplementation might be considered a potential treatment approach for PD patients with COVID-19. Despite these insights, the underlying pathophysiological mechanisms remain unclear, highlighting the vital need for further research to validate and build upon these findings.

## 1 Introduction

Coronavirus disease 2019 (COVID-19), caused by severe acute respiratory syndrome coronavirus 2 (SARS-CoV-2), has had a tremendous impact on the global economy and human health (1). As of July 28, 2024, the number of people infected with SARS-CoV-2 had reached 775,830,200 (2), and among them, the number of deaths was as high as 7,056,108 (3). Several studies have shown that patients with psychiatric diseases (PDs) are more prone to contracting COVID-19 (4, 5). Additionally, COVID-19, an illness capable of triggering systemic inflammation and a cytokine storm, is also associated with an increase in the number of diagnoses of PDs, including schizophrenia (SCZ), bipolar disorder (BD), and major depressive disorder (MDD) (6–8). These findings indicate that there may be a potential link between the pathogenesis of COVID-19 and PDs.

The involvement of inflammatory processes in both PDs and COVID-19, leading to changes in a series of indicators, such as cytokines and laboratory factors, has been consistently reported in numerous studies (8–12). For example, several hematological parameters, such as the neutrophil count (Neu#), monocyte count (Mon#), neutrophil-to-lymphocyte ratio (NLR), platelet-to-lymphocyte ratio (PLR), monocyte-to-lymphocyte ratio (MLR), and systemic immune-inflammation index (SII), are positively associated with COVID-19 (13–15), along with the levels of several biochemical parameters and cytokines, such as alanine aminotransferase (ALT), aspartate aminotransferase (AST), C-reactive protein (CRP), and interleukin 6 (IL-6) (16, 17). When inflammatory stimuli or infections occur, cytokine levels increase, causing systemic symptoms, especially during acute inflammation (9). An increase in the levels of inflammatory markers has also been observed in individuals with severe PDs such as SCZ, BD, and MDD (18, 19).

The NLR, MLR, PLR, and SII are commonly used as markers of inflammation in various diseases, such as infections and mental disorders, and these markers provide valuable prognostic insights (11, 20–25). For example, the NLR is positively correlated with patients’ self-perceived illness severity (26). Recently, an increase in the NLR was reported in patients with a first episode of psychosis (FEP), and this increase in the NLR could increase the expression of proinflammatory factors related to the stress response in these patients (27). Moreover, these ratios might be useful tools for identifying patients with BD at risk for metabolic syndrome (24, 28). On the basis of this rationale, the NLR seems to be a promising neuroinflammatory biomarker. Although these ratios lack specificity, they can assist in making treatment decisions, monitoring treatment efficacy, and predicting disease progression and complications (20, 24, 25, 29, 30).

In the study carried out by Raony et al., changes in cytokine levels were considered to be a link between immune system changes during COVID-19 and PDs, as these changes may play a role in disrupting neurotransmitter metabolism, leading to behavioral changes (31). Wang et al. reported that the levels of several interleukins, specifically IL-6, in the cerebrospinal fluid of patients with SCZ were significantly greater than those in a control group (32). Additionally, Severance et al. suggested that exposure to coronaviruses might be a concurrent risk factor for individuals with severe mental disorders (33). Furthermore, psychiatric patients with COVID-19 (PD+) require longer hospital stays and have higher rates of complications and mortality, indicating poorer outcomes (5). Therefore, the identification of indicators that are correlated with both COVID-19 and mental illness is crucial.

To date, few studies have described the hematological, serum biochemical, and cytokine parameters of patients with psychosis during and after COVID-19. Since hematological and serum biochemical analyses are the most commonly performed tests in the clinic, to determine whether other parameters from general laboratory tests have prognostic value in PD+ patients and to provide more evidence for clinical diagnosis and treatment, we investigated peripheral blood hematological and serum biochemical parameters, as well as cytokine levels, in PD+ patients.

## 2 Methods

### 2.1 Participants

One hundred four participants with PDs (83 with SCZ, 19 with BD, and 2 with MDD) and 97 healthy controls (HCs) were included in this study. PD patients who were inpatients at Xiamen Xianyue Hospital from December 1, 2020, to January 31, 2024, were recruited, and the details of the psychotropic medications they used are presented in **Supplementary Table 1**. SCZ, BD, and MDD were diagnosed according to the criteria of the Diagnostic and Statistical Manual of Mental Disorders-IV Text Revision (DSM-IV-TR). The diagnoses were confirmed by two trained psychiatrists using the Structured Clinical Interview for DSM-IV Axis I Disorders (SCID-I).

Two patient groups were included in this study: a) patients with concurrent PD and COVID-19 (PD+): This group was composed of patients hospitalized in psychiatric units. The study population comprised individuals with laboratory-confirmed SARS-CoV-2 infection, with all cases definitively diagnosed through real-time reverse transcription PCR (RT–PCR) testing of nasopharyngeal swab samples . b) Patients in the PD group and post-COVID-19 group (PD-): This study cohort comprised the PD+ patients in the post-COVID-19 recovery phase (≥30 days since symptom resolution), with confirmed SARS-CoV-2 clearance via consecutive negative RT–PCR tests and the absence of concurrent viral/bacterial infections, as validated through comprehensive microbiological screening. Blood samples from PD+ patients were collected before antiviral therapy was initiated. HCs were recruited from Xiamen Changgeng Hospital. HCs had neither current nor lifetime Axis I disorders, nor did any of their first-degree relatives have a history of such disorders. The following participants were excluded: those with concomitant major medical disorders, neurological diseases, or head injuries accompanied by loss of consciousness and/or those with substance/alcohol abuse or dependence and SARS-CoV-2 or other bacterial or viral infections.

This study was approved by the Medical Research Ethics Committee of Xiamen Xianyue Hospital in accordance with the guidelines of the Declaration of Helsinki. All participants provided written informed consent after receiving a detailed description of the study.

### 2.2 Primary outcomes

In this study, all the laboratory data, including complete blood count (XN-1000, Sysmex, Japan) and serum biochemistry data (AU5800, Beckman Coulter, USA), were retrieved from the laboratory information system (LIS). The concentrations of human serum cytokines were measured with a flow cytometer (RaiseCyte2L6C, Raisecare, Qingdao, China). PD patients were diagnosed with COVID-19 with a SARS-CoV-2 nucleic acid detection kit (fluorescent PCR method, Shanghai BioGerm Medical Biotechnology Co., Ltd.) and a nasopharyngeal swab.

### 2.3 Statistical analysis

SPSS statistics software (version 19.0) and GraphPad Prism software (version 6; 07 Graph Pad Software, Inc.) were used for statistical data analysis. Quantitative data with a normal distribution are presented as the means ± standard deviations, whereas nonnormally distributed variables are expressed as the medians (interquartile ranges, IQRs). Parametric analysis employed one-way ANOVA for group comparisons of normally distributed variables followed by Bonferroni-adjusted pairwise comparisons, whereas nonparametric analyses utilized the Mann–Whitney U test for two-group comparisons. The significance level was set at *P* < 0.05.

## 3 Results

### 3.1 Demographic and clinical characteristics

**Table 1** presents the participant demographics and clinical characteristics. All PD patients were receiving antipsychotic treatment, and the psychotropic drugs (**Supplementary Table 1**) used remained unchanged during and after COVID-19. All participants ranged in age from 14 to 88 years. There was no significant difference in the median age between the PD and HC groups (*P* > 0.05). Among the PD patients, 62 were male (59.6%), and 42 were female (40.4%). Additionally, there was no significant difference in sex distribution or body mass index (BMI) between the groups (*P* > 0.05). Because the PD patients were inpatients in a long-term care psychiatric hospital, they had not received higher education; therefore, the number of years of education of PD patients significantly differed from that of the HCs (*P* < 0.05). The average duration of COVID-19 was 10.07 days.

**Table 1:** Clinical characteristics and laboratory parameters of patients in the PD and HC groups.

Characteristics	PD (N=104)	HC (N=97)	*P*
Age, median (range)	50 (16-88)	47 (21-87)	0.0931
Gender, n (%)			0.2520
Male	62 (59.6)	50 (51.5)	
Female	42 (40.4)	47 (48.4)	
Education, years	9.11 ± 3.54	15.65 ± 3.87	<0.001
BMI (kg/m^2^)	23.64 ± 3.97	22.89 ± 1.43	0.077
Duration of COVID-19 (days) median (range)	10.07 (8-11)		

### 3.2 Hematological parameters of the PD+, PD-, and HC groups

**Table 2** shows the hematological parameters of the three groups. Compared with those in the HCs, the WBC#, Neu#, Mon#, platelet count (PLT#), NLR, MLR, PLR, SII and CRP levels in PD patients were significantly greater (*P* < 0.05). Moreover, the WBC#, Neu#, Mon#, NLR, MLR, PLR, SII and CRP levels were significantly greater (*P* < 0.05), whereas the lymphocyte count (Lym#), eosinophil count (Eos#) and basophil count (Bas#) were significantly lower (*P* < 0.05) in the PD+ group than in the other two groups. However, the Lym#, Eos#, Bas#, and PLT# values in the PD+ group were lower than those in the PD- group, whereas the Mon#, NLR, MLR, SII and CRP levels were greater in PD+ patients than in PD- patients (*P* < 0.05).

**Table 2:** Hematology findings and inflammatory markers in venous samples taken from the PD+, PD- and HC groups.

Parameters	PD+	PD-	HC	*P^+^*	*P^-^*	*P^c^*
WBC, ×10^9^/L	7.04 ± 2.74	7.02 ± 1.66	5.94 ± 1.30	<0.001	<0.001	0.955
Neu#, ×10^9^/L	4.46 ± 2.47	4.04 ± 1.30	3.15 ± 0.79	<0.001	<0.001	0.127
Lym#, ×10^9^/L	1.63 ± 0.68	2.18 ± 0.65	2.24 ± 0.52	<0.001	0.435	<0.001
Mon#, ×10^9^/L	0.85 ± 0.44	0.62 ± 0.20	0.37 ± 0.15	<0.001	<0.001	<0.001
Eos#, ×10^9^/L	0.08 ± 0.11	0.16 ± 0.13	0.14 ± 0.11	<0.001	0.243	<0.001
Bas#, ×10^9^/L	0.02 ± 0.02	0.03 ± 0.02	0.03 ± 0.02	0.001	0.676	0.004
PLT, ×10^9^/L	233.20 ± 86.14	306.90 ± 114.90	237.40 ± 50.95	0.673	<0.001	<0.001
NLR	5.58 ± 4.00	2.19 ± 0.87	1.43 ± 0.32	<0.001	<0.001	<0.001
MLR	1.75 ± 0.95	1.28 ± 0.95	0.17 ± 0.06	<0.001	<0.001	0.001
PLR	175.60 ± 121.20	154.30 ± 82.12	109.30 ± 27.92	<0.001	<0.001	0.139
SII	1286 ± 1042	673.60 ± 379.40	336.90 ± 96.97	<0.001	<0.001	<0.001
CRP (mg/l)	25.05 ± 27.35	6.00 ± 10.49	2.29 ± 1.28	<0.001	<0.001	<0.001

Data are presented as the means ± SDs. *P* values were acquired by one-way ANOVA followed by Bonferroni-adjusted pairwise comparisons. *P^+^*, comparison between the healthy control and PD+ groups. *P^-^*, comparison between the healthy control and PD- groups. *P^c^*, comparison between the PD+ and PD- groups.

WBC, white blood cell count; Neu#, neutrophil count; Lym#, lymphocyte count; Mon#, monocyte count; Eos#, eosinophil count; Bas#, basophil count; PLT, platelet count; NLR, neutrophil-to-lymphocyte ratio; MLR, monocyte-to-lymphocyte ratio; PLR, platelet-to-lymphocyte ratio; SII, systemic immune-inflammation index (platelet × neutrophil)/lymphocyte count; CRP, C-reactive protein.

### 3.3 Serum biochemical parameters of the PD+, PD-, and HC groups

**Table 3** shows the serum biochemical parameters of the three groups. The samples for biochemical testing were all collected in the morning while the participants were in a fasted state. Compared with those in the HC group, alkaline phosphatase (ALP), triglyceride (TG), serum creatine kinase (CK) isoenzyme MB (CK-MB), glucose (GLU), carbon dioxide (CO_2_), phosphate (P), and chloride (Cl) levels were greater in the PD+ group (*P* < 0.05); moreover, γ-glutamyl transferase (GGT), HDL-cholesterol (HDL-C), urea and magnesium (Mg) levels were lower in the PD+ group than in the HC group (*P* < 0.05). Compared with those in the HC group, the AST, ALP, TG, LDH, serum CK, CK-MB, GLU, CO_2_, and P levels in the PD+ group were greater (*P* < 0.05), but the GGT, cholesterol (CHO), HDL-C, urea, Mg, calcium (Ca), potassium (K) and sodium (Na) levels in the PD+ group were lower (*P* < 0.05). We found that the indicators ALT, LDL-cholesterol (LDL-C), serum creatinine (CREA), serum uric acid (UA), and Cl in the PD+ group and the indicators ALT, AST, CHO, LDL-C, LDH, CK, CREA, UA, Ca, K, and Na in the PD- group were mostly within the reference range. Furthermore, when the parameters of PD patients during and after COVID-19 were compared, the levels of AST, LDH, CK, CK-MB, CREA, and GLU in PD+ patients were significantly greater (*P* < 0.05), whereas the levels of CHO, Mg, Cl, Ca, and Na were lower (*P* < 0.05) than those in PD- patients.

**Table 3:** Biochemical test results of the PD+, PD- and HC groups.

Parameters	PD+	PD-	HC	*P^+^*	*P^-^*	*P^c^*
ALT (U/L)	27.66 ± 20.11	23.45 ± 15.97	26.57 ± 20.44	0.704	0.228	0.096
AST (U/L)	38.82 ± 31.86	22.70 ± 8.87	22.22 ± 11.60	<0.001	0.740	<0.001
GGT (U/L)	29.38 ± 21.95	25.78 ± 17.69	43.76 ± 47.30	0.006	<0.001	0.194
ALP (U/L)	85.6 3± 27.24	91.46 ± 24.19	69.51 ± 21.24	<0.001	<0.001	0.104
TG (mmol/L)	1.32± 0.48	1.41 ± 0.68	0.89 ± 0.33	<0.001	<0.001	0.259
CHO (mmol/L)	4.50 ± 0.99	4.86 ± 0.98	5.08 ± 0.94	<0.001	0.101	0.001
HDL-C (mmol/L)	1.25 ± 0.32	1.28 ± 0.33	1.45 ± 0.40	<0.001	0.001	0.533
LDL-C (mmol/L)	3.00 ± 0.83	3.15 ± 0.87	3.18 ± 0.88	0.144	0.831	0.203
LDH (U/L)	190.30 ± 67.99	168.20 ± 39.98	173.00 ± 32.24	0.024	0.345	0.005
CK (U/L)	570.10 ± 2154	108.10 ± 126.40	109.90 ± 59.94	0.037	0.898	0.030
CK-MB (U/L)	14.28 ± 15.24	10.40 ± 4.27	8.25 ± 5.29	<0.001	0.002	0.013
CREA (μmol/L)	76.11 ± 27.28	67.75 ± 31.67	74.77 ± 18.87	0.689	0.060	0.043
Urea (mmol/L)	4.13 ± 1.68	3.94 ± 1.72	4.96 ± 1.29	<0.001	<0.001	0.437
UA (μmol/L)	336.40 ± 116.30	327.10 ± 100.70	339.30 ± 78.97	0.838	0.343	0.538
GLU (mmol/L)	5.76 ± 0.13	5.31 ± 0.11	4.92 ± 0.07	<0.001	0.004	0.009
CO_2_ (mmol/L)	27.00 ± 3.08	27.27 ± 2.97	26.15 ± 2.10	0.024	0.003	0.524
P (mmol/L)	1.28 ± 0.22	1.26 ± 0.19	1.21 ± 0.14	0.012	0.048	0.495
Mg (mmol/L)	0.86 ± 0.08	0.89 ± 0.09	0.93 ± 0.08	<0.001	<0.001	0.044
Cl (mmol/L)	103.20 ± 4.04	105.30 ± 4.71	104.10 ± 2.73	0.062	0.035	<0.001
Ca (mmol/L)	2.30 ± 0.12	2.34 ± 0.12	2.36 ± 0.10	<0.001	0.694	<0.001
K (mmol/L)	4.09 ± 0.44	4.20 ± 0.43	4.20 ± 0.31	0.040	0.901	0.082
Na (mmol/L)	138.40 ± 4.18	140.80 ± 3.95	140.30 ± 2.51	<0.001	0.244	<0.001

Data are presented as the means ± SDs. *P* values were acquired by one-way ANOVA followed by Bonferroni-adjusted pairwise comparisons. *P^+^*, comparison between the HC and PD+ groups. *P^-^*, comparison between the HC and PD- groups. *P^c^*, comparison between the PD+ and PD- groups.

ALT, alanine aminotransferase; AST, aspartate aminotransferase; GGT, γ-glutamyl transferase, ALP, alkaline phosphatase, TG, triglyceride; CHO, cholesterol; HDL-C, HDL cholesterol; LDL-C, LDL cholesterol; LDH, serum lactate dehydrogenase; CK, serum creatine kinase; CK-MB, serum CK isoenzyme MB; CREA, serum creatinine; UA, serum uric acid; GLU, glucose; CO_2_, carbon dioxide; P, phosphate, Mg, magnesium; Cl, chloride; Ca, calcium; K, potassium; Na, sodium.

### 3.4 Cytokine levels in the PD+, PD-, and HC groups

**Table 4** shows the cytokine levels of the three groups. In the PD+ and PD- groups, the levels of IL-6, IL-17 and TNF-α were elevated compared with those in the HC group (*p* < 0.05), whereas the level of IL-4 was decreased (*p* < 0.05). IL-10 and interferon-gamma (IFN-γ) levels were elevated only in the PD+ group compared with those in the PD- and HC groups (*p* < 0.05). Additionally, Wilcoxon tests revealed significant increases in the levels of IL-6 and IFN-γ (*p* < 0.05) in the PD+ group compared with those in the PD- group; however, the levels of IL-17, IL-4, and TNF-α were not significantly different between the PD+ group and the PD- group (*p >* 0.05).

**Table 4:** Cytokine results of the PD+, PD- and HC groups.

Parameters	PD+ median (range)	PD- median (range)	HC Median (range)	*P^+^*	*P^-^*	*P^c^*
IL-6 (pg/ml)	6.59 (1.66-27.34)	1.66 (1.66-3.21)	1.46 (1.46-1.63)	<0.001	<0.001	<0.001
IL-10 (pg/ml)	0.96 (0.66-1.80)	0.59 (0.59-0.71)	1.27 (1.27-1.45)	<0.001	<0.001	<0.001
IFN-γ (pg/ml)	3.75 (1.36-10.29)	1.94 (0.93-4.10)	2.04 (1.67-2.61)	<0.001	0.609	<0.001
IL-17 (pg/ml)	8.16 (8.16-8.16)	8.16 (8.16-8.16)	1.90 (1.90-1.90)	<0.001	<0.001	0.102
IL-4 (pg/ml)	1.14 (1.14-1.14)	1.14 (1.14-1.14)	2.22 (2.22-2.22)	<0.001	<0.001	0.990
TNF-α (pg/ml)	1.32 (1.32-1.32)	1.32 (1.32-1.32)	1.41 (1.41-1.41)	<0.001	<0.001	0.921

Data are presented as the medians (IQRs). *P* values were acquired via the Mann–Whitney U test. *P^+^*, comparison between the HC and PD+ groups. *P^-^*, comparison between the HC and PD- groups. *P^c^*, comparison between the PD+ and PD- groups.

## 4 Discussion

This study is one of the first in which the hematological and biochemical markers and cytokine levels of hospitalized psychiatric patients with COVID-19 were evaluated. First, we detected elevated Mon# and inflammatory marker levels (NLR, MLR, SII and CRP) in these patients and PD- patients compared with HCs. Our findings are consistent with those of previous studies regarding the presence of elevated inflammatory markers in relation to COVID-19 and mental disorders (13, 22, 25, 28). Higher levels of inflammatory mediators are linked to the pathogenesis and progression of PDs, suggesting that abnormal NLRs, MLRs, and SIIs are not specific to any particular disorder but might indicate an underlying pathophysiological process leading to brain dysfunction (30, 34). These results support the theory that neuroinflammation plays a significant role in the development of PDs. Studies have suggested that patients with PDs who are experiencing acute illness may exhibit a heightened systemic response to SARS-CoV-2 infection (7, 31). However, the debate over whether inflammation triggers severe psychiatric conditions remains unresolved, and the precise mechanisms underlying such associations are yet to be fully understood. Research, including Mazza et al.’s study, revealed that the SII measured during hospitalization is positively correlated with subsequent depression and anxiety scores, indicating a potential link between systemic inflammation and the development of psychiatric symptoms (29). Wang G et al. reported an association between the risk of COVID-19 exacerbation and the levels of the inflammatory marker CRP (35), and we also demonstrated that CRP levels tended to be higher in PD+ patients.

Second, in our study, among the biochemical markers analyzed, only CK-MB and GLU levels were increased in both PD+ patients and PD- patients compared with HCs. An increase in GLU stimulates the proliferation of reactive astrocytes and promotes inflammation (36). Moreover, hyperglycemia can have profound effects on the central nervous system, thereby contributing to the pathogenesis of neuroinflammatory and neurodegenerative disorders (37, 38). In previous studies, CK-MB and GLU were found to be weak biochemical indicators of a good outcome of COVID-19 (39–42), and, consistent with our study, CK-MB and GLU levels were found to be higher in the PD+ group than in the HC group. This finding indicates the potential for viral myocarditis, cardiac injury resulting from progression to multiorgan failure (MOF), and secondary cardiac injury caused by organ-targeted pathologies (43). Among individuals with MOFs, cardiovascular diseases contribute to approximately 20% or more of life-years lost in patients with mental disorders (21). Emerging evidence indicates that common pathophysiological mechanisms exist between PDs and cardiovascular diseases, including biological, genetic, behavioral, and neurohormonal factors (44, 45). Severe inflammation and damage to multiple organs lead to lipid metabolism dysfunction and electrolyte imbalance (5, 28, 46–48). Dyslipidemia has frequently been reported in inpatients with severe mental disorders and COVID-19 (49, 50). TG, HDL-C, and LDL-C levels remained unchanged after COVID-19 in our study, which contrasts with the findings of other studies (51).

To our knowledge, we are the first to find that Mg levels are lower in both patients with COVID-19 and patients with mental disorders. Consistent with our findings, it has been reported that Mg levels decrease throughout the course of several mental disorders and COVID-19 (48, 52–54). Mg is an indispensable cation that is involved in many functions within the central nervous system, including signal transmission and intracellular signal transduction (48). Several studies have demonstrated the utility of Mg supplementation in neurological diseases and PDs (55, 56). Mg might be associated with the modulation of glutamatergic signals, which play a crucial role in neuroprotection, and Mg functions as an antagonist of NMDA receptors (48). On the basis of the available evidence, Mg supplementation might be beneficial in treating PD+ patients (52). A prompt diagnosis of electrolyte imbalance may minimize COVID-19-related morbidity and mortality.

As studies have shown that acute systemic infection, inflammatory states, and inflammation flares are accompanied by cytokine-mediated changes in plasma lipid and GLU levels (57, 58), we evaluated the cytokine levels of the three groups of participants. Additionally, according to the hypothesis of Raony et al., changes in cytokine levels can be regarded as a connection between immune system modifications and mental health disorders during COVID-19 (31). In the context of our study, significantly greater increases in IL-6 levels were observed in the PD+ and PD- groups than in the HC group. In line with previous studies, SARS-CoV-2-infected glial cells increased their secretion of IL-6 (49, 59). When the peripheral immune system is persistently activated, such as in chronic infections, autoimmune disorders, or PDs, the release of proinflammatory cytokines such as IL-6 might affect brain tissue related to neurological disorders, driving neuroinflammation and activating the neuroendocrine axis, thereby triggering depressive behaviors and cognitive impairments (32, 58). IL-6 may induce insulin resistance and increase the production of CRP in the liver by suppressing the expression of the insulin-sensitive glucose transporter in peripheral tissues (57). In line with this possibility, increased Mon# and protein levels associated with the immune response to bacterial infections have been identified in the blood of acutely ill unmedicated patients with mental disorders (32, 58). Several studies have demonstrated that high levels of IL-10 lead to poor outcomes in COVID-19 patients (59, 60), which is in line with the findings of our study. However, the role of IL-10 in psychosis is controversial. Some studies have shown no change in IL-10 in patients with SCZ or depression (58), whereas others have shown that IL-10 is elevated in patients with mental disorders (23, 58, 61). However, in our study, the level of IL-10 was decreased in PD patients, and this finding needs verification in subsequent multicenter studies with larger samples. Our study revealed that the level of IFN-γ was increased in PD patients only while they had COVID-19, suggesting that IFN-γ may be involved in the immune response after COVID-19 but not in mental illness. In addition, regardless of whether PD patients had COVID-19, the levels of TNF-α and IL-17 were greater in PD patients than in HCs, whereas the level of IL-4 was lower in PD patients than in HCs. These findings suggest that TNF-α, IL-17, and IL-4 might be related only to mental illness. However, TNF-α levels are increased in most COVID-19 patients and are correlated with the severity of the disease (59). This difference may be due to psychiatric drug therapy, which can reduce the levels of TNF-α.

The relationship between IL-17 and IL-4 in mental illness is controversial. Research has revealed that IL-17 levels are increased in individuals at ultrahigh risk of psychosis (62), whereas some studies have reported decreased IL-17 levels in BD patients (23). Consistent with the findings of Lorkiewicz et al., IL-4 levels were lower in MDD patients than in HCs (63). In addition, other studies have shown that the levels of IL-4 and IL-17 do not significantly change in patients with SCZ, BD, or MDD (58). These indicators, obtained through comprehensive collection and methodical screening, are valuable for differential diagnosis. This systematic screening method can identify significant indicators. The screened indicators suggest that PD+ patients have more severe inflammatory reactions, organ impairments, and disturbances in electrolytes and metabolism than PD- and non-COVID-19 patients do. Several mechanisms account for the neural effects of the inflammatory response. One hypothesis is that inflammatory response activation leads to increased production of proinflammatory cytokines and free radicals, potentially causing neuronal degeneration, white matter abnormalities, and decreased neurogenesis associated with the pathophysiology of SCZ (64). Cytokines can also directly modify the activity of enzymes involved in tryptophan catabolism (65). Although there are connections between immunological responses and the development of mental disorders, further exploration is needed to determine the underlying effects of immune mechanisms on brain neurochemical processes.

The limitations of our study are as follows: first, owing to the sudden onset of the COVID-19 outbreak and the fact that our hospital is a specialized psychiatric hospital, we were unable to collect data from non-PD patients with COVID-19. Second, although peripheral blood analysis provides valuable information, it is crucial to recognize the possible disparities between peripheral and central nervous system immune responses. In addition, our test results could have been affected by the fact that our samples were obtained from hospitalized PD patients on long-term antipsychotic drug therapy. Therefore, data from FEP or drug-naive patients could be collected to determine whether the findings were solely due to the use of antipsychotic drugs. Moreover, a larger sample is needed to confirm the results reported here.

## 5 Conclusion

The findings of our research indicated that both COVID-19 and psychosis can cause a cytokine storm, leading to the secretion of large amounts of IL-6. IL-6 can increase the levels of inflammatory biomarkers such as the NLR, MLR, SII, and CRP. Additionally, elevated IL-6 increases biochemical indicators such as CK-MB and GLU and decreases Mg, suggesting that both COVID-19 and psychosis affect multiple organ systems and may be related to cardiac injury, glycometabolic disorders, and electrolyte imbalance. In addition, our findings suggest that Mg supplementation might be considered a potential treatment approach for PD patients with COVID-19. A transdisciplinary approach is essential to ensure effective treatment with a focus on personalized medicine. Therefore, we suggest that both inflammatory and biochemical parameters, such as Neu#, Mon#, IL-6, NLR, MLR, SII, CRP, CK-MB, GLU, and Mg, should be monitored in PD+ patients throughout their hospitalization to improve outcomes. More research is needed to thoroughly comprehend the implications of these immune alterations throughout a longitudinal process to lay the foundation for potential progress in clinical practice.

## Data Availability

The raw data supporting the conclusions of this article will be made available by the authors, without undue reservation.
