# Supplementary material for: Hematological and biochemical markers and cytokine levels in hospitalized psychiatric patients with COVID-19
Source: Front Immunol. 2025 Apr 11;16:1536117. doi: 10.3389/fimmu.2025.1536117 (PMC12021875; doi:10.3389/fimmu.2025.1536117)
Supplement: Supplementary file 1 [file Table1.docx]

Table S1 **Psychotropic Medication Utilization in Psychiatric patients**‌‌

| PD NO. | psychotropic medications | dose(mg) |
| --- | --- | --- |
| 1 | Olanzapine | 20 |
|  | Amisulpride | 300 |
| 2 | Clozapine | 100 |
| 3 | Quetiapine | 200 |
|  | Valproic acid | 800 |
| 4 | Quetiapine | 300 |
|  | Aripiprazole | 20 |
| 5 | Quetiapine | 200 |
| 6 | Risperidone | 2 |
| 7 | Olanzapine | 10 |
| 8 | Olanzapine | 10 |
|  | Valproic acid | 1000 |
| 9 | Quetiapine | 400 |
| 10 | Amisulpride | 300 |
| 11 | Risperidone | 3 |
|  | Valproic acid | 800 |
| 12 | Risperidone | 6 |
| 13 | Duloxetine | 120 |
|  | Aripiprazole | 10 |
| 14 | Risperidone | 1 |
| 15 | Aripiprazole | 10 |
|  | Valproic acid | 1000 |
| 16 | Quetiapine | 200 |
| 17 | Risperidone | 2 |
|  | Olanzapine | 20 |
| 18 | Olanzapine | 15 |
| 19 | Clozapine | 150 |
| 20 | Olanzapine | 400 |
|  | Valproic acid | 800 |
| 21 | Clozapine | 175 |
| 22 | Risperidone | 0.5 |
| 23 | Olanzapine | 15 |
| 24 | Chlorpromazine | 300 |
| 25 | Quetiapine | 600 |
| 26 | Clozapine | 175 |
|  | Valproic acid | 1000 |
| 27 | Clozapine | 225 |
| 28 | Amisulpride | 600 |
| 29 | Clozapine | 225 |
|  | Valproic acid | 1000 |
| 30 | Quetiapine | 500 |
|  | Olanzapine | 20 |
| 31 | Olanzapine | 10 |
| 32 | Olanzapine | 10 |
| 33 | Clozapine | 200 |
| 34 | Olanzapine | 20 |
| 35 | Clozapine | 425 |
|  | Valproic acid | 1000 |
|  | Amisulpride | 800 |
| 36 | Amisulpride | 150 |
| 37 | Clozapine | 200 |
| 38 | Quetiapine | 600 |
|  | Olanzapine | 20 |
| 39 | Clozapine | 200 |
| 40 | Olanzapine | 17.5 |
|  | Aripiprazole | 30 |
| 41 | Olanzapine | 5 |
|  | Valproic acid | 500 |
| 42 | Olanzapine | 5 |
|  | Paliperidone | 6 |
| 43 | Olanzapine | 10 |
| 44 | Risperidone | 4 |
|  | Clozapine | 150 |
| 45 | Olanzapine | 10 |
| 46 | Olanzapine | 20 |
| 47 | Aripiprazole | 10 |
| 48 | Aripiprazole | 20 |
| 49 | Valproic acid | 1000 |
| 50 | Risperidone | 2 |
|  | Aripiprazole | 10 |
| 51 | Clozapine | 150 |
| 52 | Risperidone | 2 |
|  | Olanzapine | 2.5 |
| 53 | Risperidone | 5 |
|  | Aripiprazole | 10 |
| 54 | Chlorpromazine | 200 |
| 55 | Clozapine | 100 |
| 56 | Fluoxetine | 40 |
| 57 | Amisulpride | 600 |
|  | Quetiapine | 200 |
| 58 | Olanzapine | 15 |
| 59 | Risperidone | 4 |
|  | Valproic acid | 400 |
| 60 | Aripiprazole | 20 |
| 61 | Paliperidone | 3 |
|  | Amisulpride | 400 |
| 62 | Amisulpride | 550 |
| 63 | Paliperidone | 6 |
| 64 | Quetiapine | 400 |
|  | Valproic acid | 1000 |
| 65 | Quetiapine | 400 |
| 66 | Olanzapine | 12.5 |
| 67 | Risperidone | 12 |
| 68 | Risperidone | 4 |
| 69 | Risperidone | 4 |
| 70 | Valproic acid | 400 |
|  | Amisulpride | 200 |
| 71 | Clozapine | 225 |
| 72 | Risperidone | 2 |
|  | Valproic acid | 400 |
| 73 | Olanzapine | 10 |
|  | Valproic acid | 500 |
|  | Fluoxetine | 20 |
| 74 | Valproic acid | 1000 |
|  | Chlorpromazine | 400 |
| 75 | Risperidone | 6 |
|  | Clozapine | 200 |
| 76 | Olanzapine | 20 |
| 77 | Risperidone | 6 |
| 78 | Clozapine | 75 |
| 79 | Risperidone | 2 |
|  | Aripiprazole | 20 |
| 80 | Olanzapine | 20 |
|  | Risperidone | 2 |
| 81 | Olanzapine | 20 |
| 82 | Olanzapine | 5 |
| 83 | Olanzapine | 15 |
|  | Aripiprazole | 10 |
| 84 | Olanzapine | 20 |
| 85 | Paroxetine | 20 |
|  | Aripiprazole | 15 |
|  | Lurasidone | 80 |
| 86 | Chlorpromazine | 300 |
| 87 | Clozapine | 125 |
| 88 | Risperidone | 4 |
| 89 | Olanzapine | 20 |
|  | Valproic acid | 1000 |
| 90 | Aripiprazole1 | 15 |
| 91 | Paliperidone | 6 |
| 92 | Clozapine | 175 |
| 93 | Olanzapine | 20 |
|  | Risperidone | 4 |
| 94 | Olanzapine | 10 |
| 95 | Olanzapine | 15 |
|  | Valproic acid | 1200 |
| 96 | Risperidone | 2 |
| 97 | Olanzapine | 15 |
| 98 | Olanzapine | 10 |
| 99 | Amisulpride | 600 |
| 100 | Amisulpride | 300 |
|  | Clozapine | 100 |
| 101 | Aripiprazole | 10 |
|  | Fluoxetine | 40 |
| 102 | Paliperidone | 3 |
|  | Amisulpride | 700 |
| 103 | Paliperidone | 6 |
| 104 | Risperidone | 6 |
|  | Valproic acid | 1000 |
|  |  |  |

Notes:PD: psychiatry patients, NO.: number.
